# Pharmacological Properties of 2,4,6-Trihydroxy-3-Geranyl Acetophenone and the Underlying Signaling Pathways: Progress and Prospects

**DOI:** 10.3389/fphar.2021.736339

**Published:** 2021-08-31

**Authors:** Yee Han Chan, Kong Yen Liew, Ji Wei Tan, Khozirah Shaari, Daud Ahmad Israf, Chau Ling Tham

**Affiliations:** ^1^ Department of Biomedical Sciences, Faculty of Medicine and Health Sciences, Universiti Putra Malaysia, Serdang, Malaysia; ^2^ School of Science, Monash University Malaysia, Bandar Sunway, Malaysia; ^3^ Laboratory of Natural Products, Institute of Bioscience, Universiti Putra Malaysia, Serdang, Malaysia; ^4^ Department of Chemistry, Faculty of Science, Universiti Putra Malaysia, Serdang, Malaysia

**Keywords:** 2,4,6-trihydroxy-3-geranyl acetophenone (tHGA), 3-geranyl-2,4,6-trihydroxyacetophenone (3-GAP), *Melicope pteleifolia* (Champ. ex Benth.) T.G.Hartley, inflammation, asthma, allergy, signaling pathway

## Abstract

2,4,6-Trihydroxy-3-geranyl acetophenone (tHGA) is a bioactive phloroglucinol compound found in *Melicope pteleifolia* (Champ. ex Benth.) T.G.Hartley, a medicinal plant vernacularly known as “tenggek burung”. A variety of phytochemicals have been isolated from different parts of the plant including leaves, stems, and roots by using several extraction methods. Specifically, tHGA, a drug-like compound containing phloroglucinol structural core with acyl and geranyl group, has been identified in the methanolic extract of the young leaves. Due to its high nutritional and medicinal values, tHGA has been extensively studied by using various experimental models. These studies have successfully discovered various interesting pharmacological activities of tHGA such as anti-inflammatory, endothelial and epithelial barrier protective, anti-asthmatic, anti-allergic, and anti-cancer. More in-depth investigations later found that these activities were attributable to the modulatory actions exerted by tHGA on specific molecular targets. Despite these findings, the association between the mechanisms and signaling pathways underlying each pharmacological activity remains largely unknown. Also, little is known about the medicinal potentials of tHGA as a drug lead in the current pharmaceutical industry. Therefore, this mini review aims to summarize and relate the pharmacological activities of tHGA in terms of their respective mechanisms of action and signaling pathways in order to present a perspective into the overall modulatory actions exerted by tHGA. Besides that, this mini review will also pinpoint the unexplored potentials of this compound and provide some valuable insights into the potential applications of tHGA which may serve as a guide for the development of modern medication in the future.

## Introduction

2,4,6-Trihydroxy-3-geranyl acetophenone (tHGA) is a bioactive phloroglucinol compound found in *Melicope pteleifolia* (Champ. ex Benth.) T.G.Hartley, a medicinal plant with the vernacular name “tenggek burung” (Karim et al., 2011). This compound was one of the new acetophenones being identified in the leaves of the plant and the first geranyl acetophenone being reported in the plants of Rutaceae family (Shaari et al., 2006). In China and Southeast Asia countries including Malaysia, *Melicope pteleifolia* (Champ. ex Benth.) T.G.Hartley is renowned as a traditional herbal medicine for the treatment of wounds, itches, fever, rheumatism, stomach ache, trauma, abscesses, hemorrhoids, and skin diseases (Perry and Metzger, 1980; Li et al., 2011).

Similar to other *Melicope* species, major constituents of the plant are benzopyrans, acetophenones, furoquinoline and quinolinone alkaloids, and flavonoids. Although numerous studies have been done on different parts of *Melicope pteleifolia* (Champ. ex Benth.) T.G.Hartley including leaves, stems, and roots, the leaves seem to be the most valuable part of the plant as different extracts of the leaves are accountable for most of the proven pharmacological activities. Notably, tHGA was found in the methanolic extract of the plant leaves particularly young leaves as young leaves contain higher levels of secondary metabolites as well as less sugar and/or glycosidic compounds compared to mature leaves (Shuib et al., 2011).

To date, various pharmacological activities of tHGA have been discovered. These include anti-inflammatory (Shaari et al., 2011), endothelial and epithelial barrier protective (Chong et al., 2016; Sim et al., 2018; Chan et al., 2021), anti-asthmatic (Ismail et al., 2012; Lee et al., 2017a; Lee et al., 2017b; Yap et al., 2018; Ismail et al., 2019), anti-allergic (Tan et al., 2017a; Tan et al., 2017b), and anti-cancer (Cho et al., 2012a; Cho et al., 2012b) activities. Therefore, this mini review aims to summarize the reported pharmacological activities of tHGA and the underlying signaling pathways, as well as to pinpoint the potentials of this compound for drug development processes in the future.

## Pharmacological Activities of tHGA

### Anti-Inflammatory Activity

The therapeutic potential of tHGA was first discovered in 2011 when it emerged as a bioactive compound with anti-inflammatory activity in human peripheral blood mononuclear leukocytes (Human PBMLs) as revealed by bioassay-guided fractionation (Shaari et al., 2011). tHGA was found to be effective in inhibiting lipoxygenase (LOX) and cyclooxygenase (COX) enzymes *in vitro*, which are both essential in producing various inflammatory mediators such as cysteinyl leukotrienes (CysLTs) and prostaglandins (PGs) (Shaari et al., 2011; Chong et al., 2016).

The first study on the anti-inflammatory activity of tHGA *in vivo* was initiated in 2013 by using lipopolysaccharide (LPS)-induced endotoxemic BALB/c mice (Chan et al., 2021). However, tHGA was shown to be ineffective in rescuing endotoxemic mice from lethality due to its failure in suppressing the overproduction of one of the major inflammatory mediators in endotoxemia, tumor necrosis factor (TNF)-α, thus resulting in multiple organ dysfunctions (Chan et al., 2021). This finding was in line with an earlier study in 2010 which has proven that tHGA was unable to inhibit the production of nitric oxide (NO) from LPS- and interferon (IFN)-γ-stimulated RAW264.7 cells (Abas et al., 2010), possibly due to the failure of tHGA in suppressing TNF-α overproduction, since TNF-α mediates the production of NO.

As such, it is highly probable that tHGA may exert anti-inflammatory activity via significant suppression of specific inflammatory mediators such as LOX and COX enzymes (Shaari et al., 2011), but not the other common proinflammatory cytokines and mediators including TNF-α and NO (Abas et al., 2010; Chan et al., 2021). Due to its specificity, further studies on tHGA have been focusing on inflammatory disorders (Chong et al., 2016; Sim et al., 2018; Chan et al., 2021) and allergic inflammatory diseases (Ismail et al., 2012; Lee et al., 2017a; Tan et al., 2017a; Lee et al., 2017b; Tan et al., 2017b; Yap et al., 2018; Ismail et al., 2019), in which either LOX or COX plays an important role in the pathogenesis of the diseases.

### Endothelial and Epithelial Barrier Protective Activity

In the presence of proinflammatory stimuli such as LPS, toll-like receptor (TLR)-4 will be activated and a series of signaling pathways including COX pathway will be triggered. The activation of COX mediates overexpression of prostaglandin E_2_ (PGE_2_) which causes endothelial cells lining the inner vascular wall of blood vessels to lose intact, thus leading to impaired vascular integrity and endothelial hyperpermeability as a result of compromised junctional complex (Ke et al., 2017). Consequently, monocytes are capable of transmigrating across impaired endothelial barrier through cell adhesion molecules (CAMs) in the presence of monocyte chemoattractant protein (MCP)-1 (Zhang et al., 2013; Deng et al., 2019). Despite this deleterious condition, Chong et al. (2016) demonstrated that tHGA was able to inhibit monocyte adhesion which in turns suppressed their transmigration across the endothelial barrier through downregulation of CAM expression in Human Umbilical Vein Endothelial Cells (HUVECs) (Chong et al., 2016). This finding was further confirmed *in vivo* where tHGA exerted significant inhibitory effect against leukocyte transmigration across the vascular wall in LPS-induced endotoxemic BALB/c mice (Chan et al., 2021). tHGA was also discovered to significantly inhibit LPS-induced endothelial hyperpermeability in HUVECs through attenuation of cytoskeletal rearrangement (Chong et al., 2016), which was further supported by the ability of tHGA to suppress LPS-induced vascular leakage in mice (Chan et al., 2021). Interestingly, tHGA did not exert any effect on the secretion of MCP-1, a key chemokine that regulates monocyte transmigration (Chong et al., 2016). Collectively, the aforementioned findings indirectly indicate that the mode of action of tHGA might be related to its ability to suppress structural changes of endothelium instead of attenuation of inflammatory mediator secretion in the presence of LPS.

Pulmonary permeability edema, a major complication of acute lung injury (ALI), severe pneumonia and acute respiratory distress syndrome (ARDS), is associated with endothelial and epithelial barrier disruption and hyperpermeability (Lucas et al., 2009; Gonzales et al., 2015). As increased pulmonary endothelial permeability leads to epithelial hyperpermeability, tHGA which has been proven to be protective against endothelial barrier disruption was also found to inhibit epithelial barrier dysfunction in TNF-α-induced A549 cells in 2018 (Sim et al., 2018). In specific, tHGA effectively inhibited TNF-α-induced monocyte adhesion to the alveolar epithelium and subsequent transmigration as a result of downregulated MCP-1 and intercellular adhesion molecule (ICAM)-1 expression. This compound also preserved epithelial barrier integrity, as evidenced by attenuation of epithelial paracellular permeability and increment of transepithelial electrical resistance (TEER), through restoration of zonula occluden (ZO)-1, occludin, and epithelial-cadherin (E-cadherin) expression. Further investigation revealed that the epithelial barrier protective activity of tHGA was mediated by the inactivation of NF-κB, p38, and ERK MAPK pathways (Sim et al., 2018).

**FIGURE 1:**
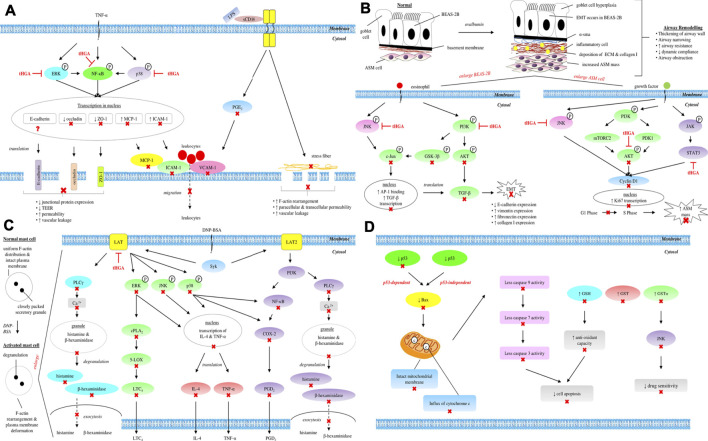
Overall mechanisms and signaling pathways underlying **(A)** endothelial and epithelial barrier protective, **(B)** anti-asthmatic, **(C)** anti-allergic, and **(D)** anti-cancer activities of tHGA. ⊣, molecular targets of tHGA; ×, downstream molecules inhibited by tHGA; ?, unknown effect.

Collectively, the findings from both LPS-induced HUVECs and TNF-α-induced A549 models confirm the effects of tHGA in protecting both endothelial and epithelial barriers, which further indicate that tHGA may be developed as a potential therapeutic agent for diseases related to endothelial and epithelial dysfunctions. The overall mechanisms and signaling pathways underlying the endothelial and epithelial barrier protective activities of tHGA are summarized and illustrated in Table 1 and Figure 1A, respectively.

**TABLE 1:** Pharmacological activities of tHGA and the key findings reported in various studies with different experimental designs.

Pharmacological activities	Experimental model; Inducer	Concentrations or doses tested	Mode of application; Route of administration	Key findings	References
Anti-inflammation	*In vitro* (Human PBML); Calcium ionophore (LOX and CysLT inhibition assay)	0.3–25 µM	Pre-treatment; N/A	- ↓ 5-LOX activity	Shaari et al. (2011)
				- ↓ LTC_4_ production	
				- ↓ PGE_2_ synthesis	
				- ↓ COX-1 and COX-2 activity with higher selectivity towards COX-2	
				- Exhibit non-redox mechanism	
	*In vivo* (5 weeks old male BALB/c mice); LPS	20–80 mg/kg	Pre-treatment; i.p	- ↔ TNF-α secretion	Chan et al. (2021)
				- ↔ histopathological changes	
				- ↔ survivability	
	*In vitro* (RAW264.7); LPS and IFN-γ	No data	Co-treatment; N/A	- ↔ NO production	Abas et al. (2010)
Endothelial and Epithelial Barrier Protection	*In vitro* (HUVEC); LPS	1.25–20 µM (5–20 µM *p* < 0.05)	Pre-treatment; N/A	- ↓ PGE_2_ secretion	Chong et al. (2016)
				- ↓ monocyte adhesion	
				- ↓ monocyte migration	
				- ↓ ICAM-1 and VCAM-1 expression	
				- ↔ MCP-1 expression	
				- ↓ paracellular and transcellular permeability	
				- ↓ F-actin rearrangement	
	*In vivo* (5 weeks old male BALB/c mice); LPS	2–100 mg/kg (2–100 mg/kg *p* < 0.05)	Pre-treatment; i.p	- ↓ vascular leakage	Chan et al. (2021)
				- ↓ leukocyte infiltration	
	*In vitro* (A549); TNF-α	3–50 µM (3–50 µM *p* < 0.05)	Co-treatment; N/A	- ↓ monocyte adhesion	Sim et al. (2018)
				- ↓ monocyte migration	
				- ↓ MCP-1 gene and protein expression	
				- ↓ ICAM-1 protein expression	
				- ↓ paracellular permeability	
				- ↓ TEER	
				- ↑ ZO-1 gene and protein expression	
				- ↑ occludin gene and protein expression	
				- ↑ E-cadherin protein expression	
				- ↓ IκBα phosphorylation	
				- ↓ p65 translocation	
				- ↓ phosphorylation of p38 and ERK MAPK	
Anti-asthma	*In vivo* (8–10 weeks old male BALB/c mice); Ovalbumin	0.2–100 mg/kg (2–100 mg/kg *p* < 0.05)	Pre-treatment; i.p	- ↓ eosinophil number and total cell count	Ismail et al. (2012)
				- ↓ infiltration of inflammatory cells and goblet cells	
				- ↓ airway hyperresponsiveness	
				- ↓ secretion of IL-4, IL-5, IL-13, and CysLTs	
				- ↓ serum IgE level	
	*In vivo* (8–10 weeks old BALB/c mice); Ovalbumin	2–20 mg/kg (2–20 mg/kg *p* < 0.05)	Pre-treatment; i.p	- ↓ eosinophil number and total cell count	Ismail et al. (2019)
				- ↓ infiltration of inflammatory cells and goblet cells	
				- ↓ airway hyperresponsiveness	
				- ↓ secretion of IL-4, IL-5, IL-10, IL-13, CysLTs, RANTES, and TGF-β	
				- ↓ serum IgE level	
	*In vivo* (6–8 weeks old female BALB/c mice); Ovalbumin	20–80 mg/kg (40–80 mg/kg *p* < 0.05)	Pre-treatment; Oral	- ↓ airway hyperresponsiveness	Lee et al. (2017a)
				- ↓ infiltration of inflammatory cells and goblet cells	
				- ↓ collagen deposition	
				- ↓ fibronectin, tenascin, and vimentin expression	
				- ↓ α-SMA expression	
				- ↓ IL-4, IL-13, and TGF-β expression	
				- ↓ IgE and IgG level	
	*In vitro* (BEAS-2B); Eosinophil	7.5–30 µM (15–30 µM *p* < 0.05)	Pre-treatment; N/A	- ↓ EMT	Lee et al. (2017b)
				- ↓ E-cadherin downregulation	
				- ↓ upregulation of vimentin, fibronectin, and collagen I	
				- ↓ TGF-β synthesis	
				- ↓ phosphorylation of JNK, PI3K, AKT, and GSK-3β	
				- ↓ c-Jun phosphorylation and translocation	
				- ↓ AP-1 binding activity	
	*In vitro* (hBSMC); Growth factor	5–20 µM (10–20 µM *p* < 0.05)	Co-treatment; N/A	- ↓ cell migration	Yap et al. (2018)
				- ↓ cell proliferation	
				- ↓ Ki67 mRNA expression	
				- ↓ cells entering S phase	
				- ↑ cell accumulation at G1 phase	
				- ↔ apoptosis	
				- ↓ cyclin D1 and p27^Kip1^ expression	
				- ↓ phosphorylation of JNK, STAT, AKT, and myr-AKT	
				- ↔ ERK, p38, mTORC2, and PDK1 phosphorylation	
Anti-allergy	*In vitro* (RBL-2H3); DNP-BSA	1.25–20 µM (5–20 µM *p* < 0.05)	Pre-treatment; N/A	- ↓ secretion of preformed mediators (β-hexosaminidase and histamine)	Tan et al. (2017a)
				- ↓ secretion of *de novo* mediators (PGD_2_, LTC_4_, IL-4, and TNF-α)	
				- ↓ mast cell degranulation	
				- ↓ cytoskeletal rearrangement	
	*In vivo* (6–8 weeks old Sprague Dawley rats); DNP-BSA	20–80 mg/kg (40–80 mg/kg *p* < 0.05)	Pre-treatment; Oral	- ↓ secretion of preformed mediators (histamine)	Tan et al. (2017a)
				- ↓ secretion of *de novo* mediators (PGD_2_, LTC_4_, IL-4, and TNF-α)	
				- ↓ mast cell degranulation	
				- ↓ morphological changes of mast cells	
	*In vitro* (RBL-2H3); DNP-BSA	1.25–20 µM (5–20 µM *p* < 0.05)	Pre-treatment; N/A	- ↓ gene expression of IL-4 and TNF-α	Tan et al. (2017b)
				- ↓ intracellular Ca^2+^	
				- ↓ phosphorylation of PLCγ, LAT, cPLA_2,_ ERK, p38, and JNK	
				- ↔ phosphorylation of Syk and PI3K	
				- ↓ 5-LOX and COX-2 expression	
				- ↓ phosphorylation and expression of IκBα	
				- ↓ p65 translocation	
				- ↔ phosphorylation of IKKα/β	
Anti-cancer	*In vitro* (MCF-7); N/A	1–20 µM (5–20 µM *p* < 0.05)	N/A; N/A	- ↓ cell viability	Cho et al. (2012a)
				- ↑ apoptosis	
				- ↑ nuclear accumulation of p53	
				- ↑ p53-Luc reporter activity	
				- ↑ Bax protein expression	
				- ↔ Bcl-2 expression	
				- ↑ cytochrome c release	
				- ↑ activation of caspase 9 and caspase 7	
	*In vitro* (MCF-7/ADR); N/A	0.1–10 µM; (2.5–10 µM *p* < 0.05)	N/A; N/A	- ↓ cell viability	Cho et al. (2012b)
				- ↑ apoptosis	
				- ↔ p53 transcriptional activity	
				- ↔ nuclear p53 expression	
				- ↑ Bax protein expression	
				- ↑ cytochrome c release	
				- ↑ activation of caspase 9, caspase 7, and caspase 3	
				- ↓ GST activity	
				- ↓ GSH level	
				- ↓ GST∏ expression	
				- ↑ sensitization to doxorubicin	
				- ↓ JNK activation	

N/A, not applicable; ↓, inhibit; ↑, increase; ↔, no effect.

### Anti-Asthmatic Activity

Asthma is a chronic pulmonary disorder initiated by airway inflammation, which paves way to irreversible airway structural remodeling (Bousquet et al., 2000; Barnes, 2008; Mims, 2015; Lange et al., 2020). Upon inhalation of allergens, increased secretion of Th2 cytokines such as interleukin (IL)-4, IL-5, IL-10, and IL-13 induces immune and structural cells to secrete various proinflammatory mediators such as CysLTs, RANTES, and transforming growth factors (TGF)-β (Chun et al., 2018), which activate the synthesis of allergen-specific immunoglobulin E (IgE) from mast cells and macrophages (Schanin et al., 2020). These series of events collectively provoke the recruitment of eosinophils and infiltration of inflammatory cells into the airways, thus leading to airway inflammation with ensuing bronchial constriction and hyperresponsiveness (Sung et al., 2021).

Previous findings of Shaari et al. (2011) proved that tHGA was a strong inhibitor of 5-LOX which drove the synthesis of CysLTs, thus alleviating airway inflammation and bronchial constriction (Shaari et al., 2011). In 2012, a follow-up study demonstrated that tHGA was able to inhibit acute allergic airway inflammation in a murine model upon single ovalbumin challenge (Ismail et al., 2012). Specifically, tHGA suppressed the production of CysLTs, IL-4, IL-5, IL-13, as well as IgE when being administered via intraperitoneal (i.p) route. These inhibitory effects subsequently contributed to the reduction of eosinophils and the suppression of inflammatory cell infiltration into the airway, thus attenuating airway hyperresponsiveness (Ismail et al., 2012).

Apart from acute allergic airway inflammation, tHGA was also proven to be effective in attenuating chronic allergic airway inflammation in 2017–2019 when it was administered orally and intraperitoneally (Lee et al., 2017a; Ismail et al., 2019). tHGA was able to counteract the overproduction of CysLTs, IL-4, IL-5, IL-13, RANTES, TGF-β, immunoglobulin G (IgG), and IgE under persistent challenge of ovalbumin. The suppression of these inflammatory mediators diminished the infiltration of inflammatory cells including eosinophils, lymphocytes, macrophages, and neutrophils into the airway, thus abrogating chronic airway inflammatory responses and hyperresponsiveness (Lee et al., 2017a; Ismail et al., 2019).

Chronic airway inflammation could lead to airway remodeling if the airway inflammatory responses are left uncontrolled or untreated (Boulet, 2018). Airway remodeling refers to irreversible structural changes of the respiratory tracts, which encompass excessive extracellular matrix (ECM) protein and collagen deposition leading to subepithelial fibrosis, increased airway smooth muscle (ASM) cell mass, goblet cell hyperplasia and hypertrophy which further contribute to mucus hypersecretion, myofibroblast accumulation, and epithelial-mesenchymal transition (EMT) (Fehrenbach et al., 2017; Banno et al., 2021). Interestingly, both oral and intraperitoneal administration of tHGA profoundly abrogated the aberrant deposition of collagen and ECM proteins such as fibronectin, tenascin, and vimentin around the airway wall, thus preventing subepithelial fibrosis (Lee et al., 2017a; Ismail et al., 2019). The expression of alpha-smooth muscle actin (α-SMA), a definitive marker of both ASM cells and differentiated fibroblasts (myofibroblasts), was also reduced significantly by tHGA (Lee et al., 2017a). Treatment of tHGA also suppressed the elevation of goblet cells, thus preventing mucus hypersecretion upon repeated ovalbumin challenge (Lee et al., 2017a; Ismail et al., 2019).

In severely remodeled airway, the epithelial cells lining respiratory tracts could lose their epithelial properties and transform into mesenchymal cells including fibroblasts and myofibroblasts in a process called EMT (Rout-Pitt et al., 2018; Yang et al., 2019). Prior to the onset of EMT, eosinophils activate multiple signaling pathways, which subsequently lead to c-Jun phosphorylation and translocation. The activated c-Jun will then increase the binding activity of activator protein (AP)-1 in the nucleus which in turns triggers the expression of TGF-β (Liu and Shang, 2020). The overproduction of TGF-β ultimately induces EMT, characterized by the downregulation of epithelial markers including E-cadherin and the upregulation of mesenchymal phenotypes such as vimentin (Liu et al., 2021). In line with the inhibitory effects of tHGA on airway remodeling proven in previous study, tHGA was found to alleviate eosinophil induced-EMT in 2017, as evidenced by the preservation of epithelial morphology, inhibition of E-cadherin downregulation, as well as the suppression of vimentin, fibronectin and collagen I upregulation in BEAS-2B human bronchial epithelial cells. The inhibitory effects of tHGA against EMT were later found to be mediated by the inhibition of TGF-β overproduction via JNK and PI3K pathways (Lee et al., 2017b).

Apart from subepithelial fibrosis, the thickening of remodeled airway can also be attributed to increased ASM mass through hyperplasia or hypertrophy (Salter et al., 2021). The study of Lee et al. (2017) proved that tHGA was capable of attenuating α-SMA expression, which is a definitive marker of ASM cells, in the presence of ovalbumin (Lee et al., 2017a). Therefore, a follow-up in 2018 further investigated the effect of tHGA on increased ASM cell mass during airway remodeling using human bronchial smooth muscle cells (hBSMCs) (Yap et al., 2018). This study demonstrated that tHGA was able to inhibit proliferation and migration of hBSMCs by reducing Ki67 and cyclin D1 expressions, as well as increasing p27^Kip1^ expression, via AKT, JNK, and STAT3 pathways (Yap et al., 2018). A summary of the overall mechanisms and signaling pathways underlying the anti-asthmatic activity of tHGA is tabulated and presented graphically in Table 1 and Figure 1B, respectively.

### Other Anti-Allergic Activities

Allergy is a hypersensitivity disorder which can cause tissue damage and life-threatening reactions including atopic dermatitis, asthma, and anaphylactic shock (Zheng et al., 2011). tHGA was first reported to contain anti-allergic activity when it significantly reduced total peripheral blood IgE and CysLTs in a murine model of allergic airway inflammation (Ismail et al., 2012). As mast cells are the major effector cells in allergic inflammation (Metcalfe et al., 1997; Kawakami and Galli, 2002) and excessive activation of mast cells by IgE results in extreme allergic inflammatory responses through the release of mediators including CysLTs (Holgate et al., 2005; Sheinkopf et al., 2008; Galli and Tsai, 2012), it indicates that the anti-allergic activity of tHGA may be exerted via mast cells.

In 2017, a study showed that tHGA attenuated IgE-mediated mast cell degranulation through the inhibition of preformed mediators (β-hexosaminidase and histamine) and *de novo* mediators (IL-4, TNF-α, prostaglandin D_2_ (PGD_2_), and leukotriene C_4_ (LTC_4_) released by dinitrophenyl (DNP)-IgE-sensitized RBL-2H3 cells (Tan et al., 2017a). The same study also reported that oral administration of tHGA in Sprague Dawley rats was able to inhibit IgE-mediated passive systemic anaphylaxis (PSA), as evidenced by the decreased serum level of histamine, PGD_2_, LTC_4_, IL-4, and TNF-α. In consistent with their *in vitro* findings, the electron microscopic examination of isolated peritoneal mast cells revealed that this compound was effective in stabilizing the structure of mast cells, therefore preventing granules from being released into the surrounding (Tan et al., 2017a).

In terms of signal transduction during IgE-mediated mast cell activation, tHGA has been reported to inhibit intracellular Ca^2+^ level and several important signaling molecules such as 5-LOX, COX-2, LAT, PLCγ1, p38, JNK, ERK, cPLA_2_, PI3K, NF-κB, IκBα, and IKKα/β (Tan et al., 2017b). Interestingly, this compound was unable to inhibit the phosphorylation of Syk and its kinase activity, which is a common molecular target for most of the mast cell stabilizers. As Syk is located upstream of LAT and PLCγ, a further investigation using siRNA knockdown assay revealed that tHGA was unable to inhibit mast cell degranulation in LAT-deficient RBL-2H3 cells (Tan et al., 2017b). As such, it was concluded that the possible molecular target for tHGA in IgE-mediated mast cell activation was the LAT protein. The overall mechanisms and signaling pathways underlying the anti-allergic activity of tHGA are summarized and illustrated in Table 1 and Figure 1C, respectively.

### Anti-Cancer Activity

tHGA (or named as 3-geranyl-2,4,6-trihydroxyacetophenone (3-GAP) in these studies) was claimed to exhibit anti-cancer activity when it was demonstrated to induce apoptosis in both adriamycin-resistant (Cho et al., 2012b) and non-resistant (Cho et al., 2012a) MCF-7 human breast cancer cells. This compound increased the nuclear accumulation and transcriptional activity of p53, which is a tumor suppressor that induces apoptotic cell death through the regulation of anti-apoptotic Bcl-2 protein family (Hemann and Lowe, 2006), in non-resistant MCF-7 cells (Cho et al., 2012a). Upon tHGA treatment, pro-apoptotic Bax protein expression was found to be increased while anti-apoptotic Bcl-2 protein level remained unchanged in both adriamycin-resistant (Cho et al., 2012b) and non-resistant (Cho et al., 2012a) MCF-7 cells. The increased Bax/Bcl-2 ratio subsequently promoted the translocation of Bax protein into mitochondrion, thus resulting in increased mitochondrial permeability. The permeabilized mitochondrial membrane then allowed the release of cytochrome c into cytosol, followed by activation of caspases, which serve as the initiator of caspase cascade and executioner protease in apoptosis signaling (Cho et al., 2012a). Interestingly, these studies have collectively proven that tHGA did not only induce apoptosis in non-resistant MCF-7 cells via Bax-mediated mitochondrial pathway (Cho et al., 2012a), but was also able to induce apoptosis in adriamycin-resistant MCF-7 cells via p53-independent caspase-3 activation pathway (Cho et al., 2012b).

Cancer cells might progressively develop multi-drug resistance (Lage, 2008) due to overexpression of a detoxification enzyme, namely glutathione S-transferase (GST) (Traverso et al., 2013; Pljesa-Ercegovac et al., 2018). Gratefully, tHGA was demonstrated to be an effective GST inhibitor which restored the sensitization of cancer cells to various anti-cancer drugs, as proven by the stark elevation of sensitivity of adriamycin-resistant MCF-7 cells to doxorubicin upon tHGA treatment (Cho et al., 2012b). Apart from drug resistance, resistance to oxidative stress could also be a challenge in anti-cancer therapy. In order to cope with higher oxidation state, cancer cells develop enhanced antioxidant system such as increased GSH level (Lv et al., 2019). Fortunately, tHGA was able to deplete GSH level, thereby reducing the antioxidant capacity of the cancer cells. The restoration of drug sensitivity and oxidative capacity were dictated by indirect inhibition of JNK through the repression of GSTΠ (Cho et al., 2012b). A summary of overall mechanisms and signaling pathways underlying the anti-cancer activity of tHGA is presented and illustrated in Table 1 and Figure 1D, respectively.

## The Way Forward

Being the first geranyl acetophenone reported in the plants of Rutaceae family (Shaari et al., 2006) with proven anti-inflammatory (Shaari et al., 2011), endothelial and epithelial barrier protective (Chong et al., 2016; Sim et al., 2018; Chan et al., 2021), anti-asthmatic (Ismail et al., 2012; Lee et al., 2017a; Lee et al., 2017b; Yap et al., 2018; Ismail et al., 2019), anti-allergic (Tan et al., 2017a; Tan et al., 2017b), and anti-cancer (Cho et al., 2012a; Cho et al., 2012b) activities, tHGA is deemed to be a novel compound with several advantages that make it a promising candidate to be further developed into a potential drug lead by the current pharmaceutical industry.

Firstly, the specificity of tHGA in terms of molecular target holds potential as a promising anti-asthma remedy. As of now, glucocorticosteroids remain the mainstay of asthma therapy (Beckett and Howarth, 2003) despite that glucocorticosteroids have been reported to be less effective in abrogating airway remodeling and hyperresponsiveness (Doerner and Zuraw, 2009; Royce and Tang, 2010; Baraket et al., 2012). Remarkably, apart from the inhibitory effect on airway inflammation, tHGA drastically alleviates airway remodeling, mainly through the suppression of EMT and attenuation of increased ASM cell mass (Ismail et al., 2012; Lee et al., 2017a; Lee et al., 2017b; Yap et al., 2018; Ismail et al., 2019). As such, the dual inhibitory effects of tHGA would be of great value for its further development into a potential anti-asthmatic drug at clinical level.

Secondly, mast cells stabilizers that are clinically used to alleviate allergic responses such as ketotifen fumarate often target Syk (Braselmann et al., 2006; Norman, 2011; Krause et al., 2013; Simmons, 2013; Harvima et al., 2014). Notably, Syk does not only act as an upstream modulator that signals mast cell degranulation and cytokine gene transcription in the manifestation of allergic responses, but it is also required for the development and function of various tissues (Saitoh et al., 2000). Therefore, inhibiting Syk may produce unwanted side effects and adverse drug reactions (Krause et al., 2013). Noteworthily, tHGA specifically targets LAT which plays a vital role in FcεRI-mediated signaling and effector function in mast cells, while not having any obvious role in mast cell development (Saitoh et al., 2000; Tan et al., 2017b). Therefore, this compound may have the potential to be further developed into a drug candidate in treating IgE-mediated mast cell degranulation in allergic events.

Thirdly, the findings of absorption, distribution, metabolism, excretion, and toxicity (ADMET) analysis have proven that tHGA exhibited good intestinal absorption, good aqueous solubility, moderate blood-brain barrier penetration, cytochrome P450 2D6 (CYP_2_D_6_) inhibition, and no hepatotoxicity (Ng et al., 2018). Also, toxicology study of tHGA via toxicity prediction by computer assisted technology (TOPKAT) further revealed that tHGA was biodegradable, non-mutagenic, non-carcinogenic, skin non-irritant, and ocular non-irritant (Ng et al., 2018). Taken together, these computational findings strongly validate that tHGA is a safe drug candidate that has the potential to be further developed as a modern medication for treating various diseases. It is important to note that findings from a pharmacokinetic study on tHGA has demonstrated that the oral bioavailability of tHGA in mice was not high (Alkhateeb et al., 2020). However, liposomal formulation has successfully improved the relative oral bioavailability of tHGA from 9.1 to 21.0% which was a 2.3-fold increment. Despite having low oral bioavailability, the therapeutic activity of tHGA has been well demonstrated in pre-clinical trials, which indirectly reflect the efficacy of this compound. Also, animal-to-human dose translation of tHGA results in doses that are considerably optimum and applicable for human consumption. In accordance to the body surface area (BSA) normalization method approved by the Food and Drug Administration (FDA), minimum effective dose of 40 mg/kg in mice corresponds to human equivalent dose (HED) of 3.24 mg/kg (Reagan et al., 2008). However, it is highly recommended that tHGA should be further developed into a potential therapeutic remedy through the incorporation of nanoformulation technology.

Fourthly, from the economical and practicability aspects, tHGA with 99% purity can be synthesized chemically via direct C-alkylation by using 2,4,6-trihydroxyacetophenone and geranyl bromide as the starting materials (Ismail et al., 2012). As conventional extraction methods are time-consuming and labor-intensive, the ability to chemically synthesize tHGA in large scale has greatly overcome the challenges in isolating and purifying tHGA from the complex methanolic extracts, thereby increasing its potential and capacity to be developed as a drug lead in the current pharmaceutical industry. In the accelerating pharmaceutical industry, the capability to chemically synthesize drug analogs with enhanced pharmacological efficacy could greatly increase the potential of a drug candidate. tHGA is a drug-like compound consisting of bioactive phloroglucinol structural core with hydrophilic acyl group and hydrophobic geranyl group. The potent pharmacological activities of tHGA particularly its inhibitory effect against LOX are shown to be contributed by the geranyl group that is lipophilic in nature (Ng et al., 2014). As acylphloroglucinol groups have command of many biological properties, the modifications of acyl substituents while preserving the lipophilic geranyl group are believed to further enhance the pharmacological efficacy of a drug candidate (Ng et al., 2014). As expected, several analogs of tHGA with different acyl moieties exhibited better LOX inhibitory effect in comparison to the parent compound (Ng et al., 2014). Notably, the elongation of aliphatic chain length and the introduction of aromatic moiety have drastically enhanced LOX inhibitory effect as a result of increased lipophilicity (Kubo et al., 2013). These improvements greatly warrant tHGA to be further developed into a clinical drug in treating inflammatory disorders.

To date, there have been quite a number of *in vitro* and *in vivo* studies investigating the pharmacological activities of tHGA on inflammation (Shaari et al., 2011), endothelial and epithelial barrier dysfunctions (Chong et al., 2016; Sim et al., 2018; Chan et al., 2021), asthma (Ismail et al., 2012; Lee et al., 2017a; Lee et al., 2017b; Yap et al., 2018; Ismail et al., 2019), and allergy (Tan et al., 2017a; Tan et al., 2017b). On the other hand, the anti-cancer activity of tHGA is relatively less well studied and has been limited to *in vitro* approaches (Cho et al., 2012a; Cho et al., 2012b). Therefore, several potential research directions are suggested below for further investigations on the anti-cancer activity of tHGA in the future. As reported by previous studies, the abilities of tHGA to mediate mitochondrial dysfunction and reduce GSH level have collectively induced the apoptosis in MCF-7 human breast cancer cells (Cho et al., 2012a; Cho et al., 2012b). As GSH regulates the homeostasis of reactive oxygen species (ROS) and higher concentration of ROS leads to accumulation of oxidative stress and provokes mitochondrial toxicity (Kennedy et al., 2020), thus the effect of tHGA on the production of ROS should be further examined. In cancer cells, a natural compound is deemed to be efficacious if it is capable of reducing GSH level as low GSH level could decrease the antioxidant capacity of cancer cells, thereby inducing cancer cell apoptosis. Inversely, in normal cells, a natural compound is expected to increase GSH level in order to boost the antioxidant capacity against oxidative stress induced by various oxidative attacks such as xenobiotics. It is important to highlight that current studies of tHGA on apoptosis were limited to *in vitro* model of cancer cells (Cho et al., 2012a; Cho et al., 2012b) and the effect of tHGA on xenobiotic-induced apoptosis in normal cells remains unknown. Rutin, a bioactive plant flavonoid and natural antioxidant, has been well reported to induce apoptosis in various cancer cells including MCF-7 human breast cancer cells (Iriti et al., 2017; Hasani et al., 2018; Saleh et al., 2018; Kunjiappan et al., 2019; Haifeng et al., 2020). Similar to tHGA, rutin exhibited its pro-apoptotic effect in MCF-7 cells by increasing Bax expression, GSH level, caspase cascade activation and chemosensitivity, as well as decreasing Bcl-2 expression (Iriti et al., 2017; Hasani et al., 2018; Saleh et al., 2018; Kunjiappan et al., 2019; Haifeng et al., 2020). However, in contrast to tHGA, rutin was capable of inhibiting mercury-induced apoptosis in normal cells (Caglayan et al., 2019). Specifically, rutin increased GSH level and activities of antioxidant enzymes (catalase, glutathione peroxidase, and superoxide dismutase), as well as decreased malondialdehyde level (Caglayan et al., 2019). As it has been demonstrated that rutin was not only able to induce pro-apoptotic effect in cancer cells via similar mechanisms as tHGA (Iriti et al., 2017; Hasani et al., 2018; Saleh et al., 2018; Kunjiappan et al., 2019; Haifeng et al., 2020), but also capable of inhibiting xenobiotic-induced apoptosis in normal cells (Caglayan et al., 2019), therefore it is probable that tHGA may be able to suppress xenobiotic-induced apoptosis in normal cells as well. As such, it would be worthwhile to investigate the effect of tHGA on xenobiotic-induced apoptosis in normal cells for future studies. In signal transduction process, NF-κB is a member of transcription family which regulates various physiological processes including inflammation, cell apoptosis, cell migration, and cell cycle (Thornburg et al., 2003). Upon activation of NF-κB pathway, the secretion of various proinflammatory cytokines such as IL-4, IL-5, and IL-13 will be upregulated, thus leading to overwhelming inflammatory responses. Although NF-κB could be activated by various proinflammatory stimuli, the regulatory effects of tHGA on NF-κB activation and proinflammatory cytokine secretion were limited to TNF-α (Sim et al., 2018), DNP-BSA (Tan et al., 2017a; Tan et al., 2017b), and ovalbumin-induced (Ismail et al., 2012; Lee et al., 2017a; Ismail et al., 2019) models thus far. It would be interesting to further investigate the effects of tHGA on NF-κB pathway as well as the secretion of various proinflammatory cytokines induced by other proinflammatory stimuli. As such, LPS that has been widely reported to activate NF-κB pathway and trigger proinflammatory cytokine secretion was chosen as the inducer in one of our ongoing studies to examine the effects of tHGA on endothelial dysfunction. Besides that, 2,4-dinitrochlorobenzene (DNCB), an inducer that has been widely used to induce skin inflammation, was also tested in one of our ongoing studies to examine the effects of tHGA on the secretion of various proinflammatory cytokines in a murine model of atopic dermatitis. It is believed that the findings obtained from all these studies using various proinflammatory stimuli will help to provide a better insight into the potentials of tHGA to be developed as a clinical drug in the future.

## Conclusion

In short, tHGA exhibits significant pharmacological activities against inflammation, endothelial and epithelial barrier dysfunctions, asthma, allergy, and cancer through the modulatory actions on specific molecular targets. Notably, tHGA is safe for consumption, capable of being chemically synthesized, has potentials for nanoformulation and analog synthesis, possesses targeted action on LAT, as well as exerts dual inhibitory effects on airway inflammation and remodeling processes. These beneficial characteristics have collectively enhanced the drug-like properties and pharmacological efficacy of tHGA, thereby strengthening its potential and capability as a drug lead in the current pharmaceutical industry. As such, we sincerely hope that this mini review could provide some useful insights into the potential applications of tHGA which may serve as a guide for future drug development processes.
